# Management of Fournier’s gangrene during the Covid-19 pandemic era: make a virtue out of necessity

**DOI:** 10.1186/s12610-022-00162-y

**Published:** 2022-07-19

**Authors:** Alessio Paladini, Giovanni Cochetti, Angelica Tancredi, Matteo Mearini, Andrea Vitale, Francesca Pastore, Paolo Mangione, Ettore Mearini

**Affiliations:** Department of Medicine and Surgery, Urology Clinic, University of Perugia, 06129 Perugia, Italy

**Keywords:** Fournier’s gangrene, Necrotizing fasciitis, Urologic emergency, Surgical debridement

## Abstract

**Background:**

Fournier’s gangrene (FG) is a necrotizing fasciitis caused by aerobic and anaerobic bacterial infection that involves genitalia and perineum. Males, in their 60 s, are more affected with 1.6 new cases/100.000/year. Main risk factors are diabetes, malignancy, inflammatory bowel disease. FG is a potentially lethal disease with a rapid and progressive involvement of subcutaneous and fascial plane. A multimodal approach with surgical debridement, antibiotic therapy, intensive support care, and hyperbaric oxygen therapy (HBOT) is often needed.

We present the inpatient management of an FG case during the Covid-19 pandemic period. A narrative review of the Literature searching “Fournier’s gangrene”, “necrotizing fasciitis” on PubMed and Scopus was performed.

**Case presentation:**

A 60 years old man affected by diabetes mellitus, with ileostomy after colectomy for ulcerative colitis, was admitted to our Emergency Department with fever and acute pain, edema, dyschromia of right hemiscrotum, penis, and perineal region. Computed tomography revealed air-gas content and fluid-edematous thickening of these regions. Fournier’s Gangrene Severity Index was 9. A prompt broad-spectrum antibiotic therapy with Piperacillin/Tazobactam, Imipenem and Daptomycin, surgical debridement of genitalia and perineal region with vital tissue exposure, were performed. Bedside daily surgical wound medications with fibrine debridement, normal saline and povidone-iodine solutions irrigation, iodoform and fatty gauze application, were performed until discharge on the 40^th^ postoperative day. Every 3 days office-based medication with silver dressing, after normal saline and povidone-iodine irrigation and fibrinous tissue debridement, was performed until complete re-epithelialization of the scrotum on the 60^th^ postoperative day.

**Conclusions:**

FG is burdened by a high mortality rate, up to 30%. In the literature, HBOT could improve wound restoration and disease-specific survival. Unfortunately, in our center, we do not have HBOT. Moreover, one of the pandemic period problems was the patient’s displacement and outpatient hospital management. For all these reasons we decided for a conservative inpatient management. Daily cleaning of the surgical wound allowed to obtain its complete restoration avoiding surgical graft and hyperbaric oxygen chamber therapy, without foregoing optimal outcomes.

## Résumé

**Contexte:**

La gangrène de Fournier (GF) est une fasciite nécrosante causée par une infection bactérienne aérobie et anaérobie qui implique les organes génitaux et le périnée. Les hommes, dans la soixantaine, sont plus touchés avec 1,6 nouveau cas/100 000/an. Les principaux facteurs de risque sont le diabète, les tumeurs malignes, et les maladies inflammatoires de l’intestin. La GF est une maladie potentiellement mortelle avec une atteinte rapide et progressive du plan sous-cutané et fascial. Une approche multimodale, avec débridement chirurgical, antibiothérapie, soins de soutien intensif et oxygénothérapie hyperbare (OHB), est souvent nécessaire. Nous présentons la prise en charge en milieu hospitalier d’un cas de GF pendant la période de pandémie de Covid-19. Une revue narrative de la littérature, recherchant «gangrène de Fournier», «fasciite nécrosante» sur PubMed et Scopus, a été réalisée.

**Cas clinque:**

Un homme de 60 ans, atteint d’un diabète sucré et porteur d’une iléostomie après colectomie pour colite ulcéreuse, a été admis dans notre service d’urgences, avec fièvre et des douleurs aiguës, œdème et dyschromie de l’hémiscrotum droit, du pénis et de la région périnéale. La tomodensitométrie a révélé une teneur en air-gaz et un épaississement fluide-œdémateux de ces régions. L’indice de gravité de la gangrène de Fournier était de 9. Une antibiothérapie rapide à large spectre avec Pipéracilline/tazobactam, imipénème et daptomycine, et un débridement chirurgical des organes génitaux et de la région périnéale avec exposition des tissus vitaux, ont été effectués. Ont été réalisés au chevet du patient, un traitement quotidien des plaies chirurgicales, avec débridement de la fibrine, irrigation par solution saline normale et solution de povidone-iode, et application de gaze iodoforme et grasse, jusqu’à la décharge au 40^ème^jour postopératoire. Tous les 3 jours, un traitement à base de médicaments d’officine avec pansement à l’argent a été réalisé après irrigation par solution saline normale et solution de povidone-iode, et débridement de la fibrine des tissus, jusqu’à la ré-épithélialisation complète du scrotum au 60^ème^ jour postopératoire.

**Conclusions:**

La GF est grevée d’un taux de mortalité élevé, jusqu’à 30%. Dans la littérature, l’OHB pourrait améliorer la restauration des plaies et la survie spécifique de la maladie. Malheureusement, dans notre centre, nous n’avons pas d’OHB. En outre, l’un des problèmes de la période pandémique était le déplacement du patient et la prise en charge ambulatoire des hôpitaux. Pour toutes ces raisons, nous avons opté pour une prise en charge conservatrice en milieu hospitalier. Le nettoyage quotidien de la plaie chirurgicale a permis d’obtenir sa restauration complète en évitant la greffe chirurgicale et la thérapie en chambre à oxygène hyperbare, sans renoncer à des résultats optimaux.

**Mots-clés:**

Gangrène de Fournier, fasciite nécrosante, urgence urologique, débridement chirurgical.

## Background

The Fournier’s gangrene (FG) is a necrotizing fasciitis caused by polymicrobial aerobic and anaerobic bacterial infection that involves genitalia and perineum [1]. Males, in their 60 s, are more affected with 1.6 new cases/100.000/year and the male:female ratio is 10:1. Main recognized risk factors are states of immune system impairment as oldness, alcohol and tobacco consumption, cardiovascular diseases, renal and liver impairment, diabetes mellitus, malignancy and inflammatory bowel disease [2–5].

FG is a potentially lethal disease with a rapid and progressive involvement of the skin, the subcutaneous fat tissue until fascial planes. Inflammation and oedema lead to obliterating endarteritis with thrombosis of blood subcutaneous vessels and consequent ischemia and necrosis along dartos fascial, Colle’s fascia, Scarpa’s fascia and abdominal wall [6].

FG is a potentially lethal condition with a high mortality rate of 20–30% [7]. The standard of care is a prompt multimodal approach including intravenous fluid resuscitation, broad-spectrum antibiotic therapy, surgical extensive debridement and successive wound cares [8, 9]. In this aggressive disease the time is gold.

In order to improve the knowledge on the field, we describe a case of a male affected by several predisposing conditions at high risk of death for FG, immediately treated with a successful multimodal approach during the Covid-19 pandemic period.

A narrative review of the literature was performed on PubMed and Scopus using as researching terms “Fournier’s gangrene” and “necrotizing fasciitis”. All the available English language full-text original article, case series, case report of interest, published from January 2013 until December 2021, were reported in the Table 1 [10–198]. Review articles, meeting reports and congress poster and abstracts were all excluded.

**Table 1:** Narrative review of the literature about fournier’s gangrene

Reference	Year	Gender	N. of cases	Mean age	Surgical debridement	Days of hospital stay	Sepsi / ICU	Hyperbaric oxygen therapy	Pathogen	N. of death
Bensardi FZ et al. [10]	2021	70 M, 14 F	84	49	ND	13	ND	0	ND	6
Vargo E et al. [11]	2021	M	1	64	1	9	0	0	ND	0
Trama F et al. [12]	2021	M	1	56	1	ND	0	1	Escherichia coli, Bacteroides caccae	0
Elahabadi I et al. [13]	2021	M	1	25	1	30	1	ND	ND	0
De La Torre M et al. [14]	2021	M	1	24	1	24	1	ND	Streptococcus pyogenes (Group A)	0
Winyard JC et al. [15]	2021	M	1	16	1	ND	ND	ND	ND	0
Gul MO et al. [16]	2021	13 M, 9 F	22	56.7 ± 12.1	2.7 ± 2.4	24.1 ± 18.9	10	ND	E. Coli(5) + S. aureus (1)/ Proteus (1)/ + Corynebacterium (1)/ + Enterococcus (1)/ + Acinetobacter (2), P. Mirabilis(1), A. baumannii (1), P. Anaerobium (1), K. pneumoniae + Acinetobacter (1), S. Agalactie (1), E. faecium (3), S. Epidermidis (1), B. fragilis (1), Pseudomonas + E. Faecium (1)	6
Rivera-Alvarez F et al. [17]	2021	M	1	65	1	ND	ND	ND	E. Coli, E. Faecalis, and Bacteroides species	ND
Michalczyk Ł et al. [18]	2021	M	35	58	3 (13) 2 (22)	26 (13) 23 (22)	ND	13	E. Coli, P. Aeruginosa, E. Faceais	4
Moon JY et al. [19]	2021	M	1	66	2	15	1	0	ND	0
Lahouar R et al. [20]	2021	M	1	35	1	15	1	ND	S. Aureus	0
Shah T et al. [21]	2021	M	1	62	1	17	0	0	ND	0
Tsuge I et al. [22]	2021	M	1	64	3	ND	0	0	E. tarda and S. anginosus, E. Coli, E. Faecalis	0
Duarte I et al. [23]	2021	M	1	65	1	ND	1	0	E. Coli, E. Faecalis, K. Pneumoniae, P. Mirabilis, C.albicans	1
Wong R et al. [24]	2021	65 M, 14F	79	60	1 (62), 2 (17)	5	13	ND	ND	13
Beecroft NJ et al. [25]	2021	33 F, 110 M	143	55 F, 53.5 M	2	11 (M), 13 (F)	ND	ND	Gram positive, gram negative, fungal	2 F, 8 M
Oyelowo N et al. [26]	2021	M	31	60 ± 12	1–2 (24), 3–4 (5), > 4 (2)	15 (2), 20–30 (19), 35–42 (8), > 42(2)	4	ND	Polymicrobial flora (most common: E. coli)	3
Kundan M et al. [27]	2021	M	1	50	> 1	ND	ND	ND	ND	0
Parkin CJ et al. [28]	2021	M	1	51	> 2	20	1	ND	ND	0
Grabińska A et al. [29]	2021	M	1	60	> 1	46	1	ND	E. Coli, P. Aeruginosa	0
Sahra S et al. [30]	2021	M	1	45	2	ND	0	0	A. schaalii	0
Provenzano D et al. [31]	2021	M	1	66	3	20	ND	0	E. coli	0
Elbeddini A et al. [32]	2021	F	1	71	4	14	ND	ND	Gram-positive cocci (*S. anginosus)*, bacilli Gram-negative, Gram-positive	0
Kostovski O et al. [33]	2021	F	1	59	2	35	1	ND	ND	0
El Hasbani G et al. [34]	2021	M	1	69	1	ND	ND	0	K. pneumoniae, C. albicans	1
Voordeckers M et al. [35]	2020	M	1	53	2	ND	ND	0	*P. aeruginosa*	1
Sihombing AT et al. [36]	2020	M	1	80	2	ND	1	ND	ND	1
Maghsoudi LH et al. [37]	2020	M	1	30	1	21	ND	ND	ND	0
Zhang N et al. [38]	2020	10 M, 2 F	12	60	ND	ND	3	10	E. coli, P.aeruginosa, E. Faecalis, S.aureus, Acinetobacter	1
Rakusic Z et al. [39]	2020	M	1	76	3	49	ND	ND	P. mirabilis, P. aeruginosa, E. faecalis	1
Kasbawala K et al. [40]	2020	F	1	37	6	28	1	ND	ND	0
Barone M et al. [41]	2020	M	1	80	1	7	1	ND	ND	0
Batmaz O et al. [42]	2020	M	1	70	3	ND	1	ND	Klebsiella pneumoniae spp	1
Syllaios A et al. [43]	2020	M	1	66	3	25	ND	1	S. anginosus, S. aureus e C. koserii	0
Padilla ME et al. [44]	2020	M	1	5	1	56	ND	1	S. Marcences	0
Creta M et al. [45]	2020	152 M, 9 F	161	66.5 ± 15.2	139	ND	ND	72	ND	46
Hatipoglu E et al. [46]	2020	31 M, 4 F	35	58.14 ± 12.71	> 1	ND	12	2	ND	2
Elbeddini A et al. [47]	2020	M	1	72	3	30	ND	1	Bacteroides ovatus, Prevotella denticola e Actinomyces species	0
Ellegård L et al. [48]	2020	F	1	52	4	18	1	1	Mixed flora (aerobi e anaerobi)	0
Lindsay PJ et al. [49]	2020	M	1	51	6	30	1	ND	ND	0
Hyun DW et al. [50]	2020	M	1	62	> 3	84	1	1	ND	0
Dowd K et al. [51]	2019	M	1	43	2	ND	1	0	ND	0
Del Zingaro M et al. [52]	2019	M	1	52	1	17	0	ND	S.lugdunensis	0
Zhang C et al. [53]	2019	13 M 3 F	16	30–76	1	29.6	ND	16	ND	0
Del Zingaro M et al. [6]	2019	M	1	76	1	ND	0	1	P. Putita, S. Maltophilia, S. Haemolyticus, S. Warneri	0
Amin A et al. [54]	2019	M	1	45	4	40	1	ND	S. aureus, F. magna, C. amycolatum	0
Nagano Y et al. [55]	2019	M	1	34	1	41	0	ND	Staphylococcus aureus (MRSA)	0
Kus NJ et al. [56]	2019	F	1	84	1	ND	1	1	Mixed flora, A. europaeus and A. schaalii	0
Rodler S et al. [57]	2019	M	1	39	2	27	1	1	Peptostreptococcus anaerobius, C. Albicans	0
Çalışkan S et al. [58]	2019	35 M 1 F	36	59.27 ± 12.91	> 1	19 ± 10.44	ND	ND	E. coli (1), E.coli e Corynebacterium (2), E.coli e C. albicans (2), A. turicensis (1), B. fragilis (1), S.aureus (MRSA. 2)	1
Magdaleno-Tapial J et al. [59]	2019	M	1	38	2	ND	ND	ND	ND	ND
Joury A et al. [60]	2019	M	1	51	1	ND	1	1	S. aureus (MRSA), Edwardsiella tarda, K. oxytoca, anaerobic Gram-negative bacteria, Prevotella	ND
Sparenborg JD et al. [61]	2019	41 M 1 F	42	53.45	3.2	19.6	11	ND	ND	3
Elshimy Get al. [62]	2019	M	1	57	2	ND	ND	1	ND	ND
Lin HC et al. [63]	2019	56 M 4 F	60	53.0 ± 15.9	1 (51), 2(8), 3(1)	ND	ND	2	E. Coli, E. Faecalis, P. Mirabilis, K. Pneumoniae, Peptostreptococco, P. Aeruginosa	1
Rachana K et al. [64]	2019	M	1	50	1	18	0	ND	E. Coli, B. Fragilis, **F. varium,** P.aeruginosa	0
Louro JM et al. [65]	2019	14 M, 1F	15	66.9	3.3	46.8	ND	ND	mixed flora (7), negative results (2). MO found: *S.aureus, E.faecalis, E. coli, A. baumannii, P. aeruginosa, S.pyogenes*, *E. faecium, E. cloacae, K. pneumoniae, S.epidermidis, B. fragilis, Corynebacterium, Candida albicans, A.fumigatus*. multidrug resistant *S.aureus (1)*	ND
Escobar-Vidarte MF et al. [66]	2019	F	1	80	1	ND	ND	1	ND	0
Onder CE et al. [67]	2019	M	1	64	3	30	ND	ND	ND	0
Heijkoop B et al. [68]	2019	ND	14	ND	6	36	8	3	ND	1
Mostaghim A et al. [69]	2019	M	1	38	1	ND	0	1	*E. coli, E. faecalis, Bacteroides thetaiotaomicron, S. agalactiae, Clostridium clostridioform*, Gram-positive bacilli e cocci	0
Zhou Z et al. [70]	2019	M	1	58	1	ND	1	ND	ND	0
Majdoub W et al. [71]	2019	F	1	70	0	0	1	0	E. Coli, Bacteroides spp	1
Aslan N et al. [72]	2019	M	1	12	1	8 h	1	0	P. Aeruginosa	1
AlShehri YA et al. [73]	2019	M	1	58	1	60	ND	1	ND	0
Moussa et al. [74]	2019	M	1	58	1	18	0	0	*S. aureus, E. coli*	0
Hahn et al. [75]	2018	33 M 11F	44	54.4	3.3	47	18	ND	Polymicrobial flora (Escherichia coli, Enterococcus, Staphylococcus, Klebsiella) (7), Monomicrobial flora (Staphylococcus, Escherichia coli, Klebsiella, Streptococcus, Enterococcus, Candida) (22)	9
Overholt et al. [76]	2018	M	1	44	2	13	0	0	Escherichia coli, Enterococcus avium, Gemella morbillorum	0
Pehlivanli et al. [77]	2018	19 M 4F	23	65.9	6	18	ND	ND	Escherichia coli, Klebsiella, Staphylococci, Enterobacter	5
Kranz et al.[78]	2018	154 M	154	62.7	4.2	26.6	104	13	mixed flora (73), Streptococci (12), Staphylococci (10), Enterococcus (10), Citrobacter (1), Pseudomonas (1), Candida (2)	17
Kobayashi et al. [79]	2018	M	1	68	1	59	1	0	Escherichia coli	0
Pandey et al. [80]	2018	M	1	65	1	ND	ND	ND	ND	ND
Matsuura et al. [81]	2018	M	1	88	ND	ND	ND	0	ND	1
Sen et al. [82]	2018	M	1	47	1	18	0	0	Rhizobium radiobacter	0
Elsaket et al. [83]	2018	43 M 1F	44	51	1.33	26	6	ND	Staphylococcus aureus, Acinetobacter, Streptococcus pyogenes, Proteus mirabilis,	5
Takano et al. [84]	2018	F	1	44	1	ND	ND	0	Streptococcus constellatus, Clostridium ramosum	1
Semenič et al. [85]	2018	M	1	30	2	16	1	0	Escherichia coli, Bacteroides fragilis, Prevotella oralis, Streptococcus anginosus	0
Abbas-Shereef et al. [86]	2018	M	1	71	> 1	30	1	0	Pseudomonas aeruginosa, Klebsiella pneumoniae, Candida albicans, Staphylococci, Group A Streptococcus	0
Wetterauer et al. [87]	2018	20 M	20	66	4	ND	15	0	Escherichia coli, Klebsiella, Pseudomonas aeruginosa	3
Demir et al. [88]	2018	49 M 25F	74	57.6	1.87	23.18	ND	ND	Escherichia coli, Staphylococcus aureus, Streptococci, Enterobacter, Pseudomonas aeruginosa, Bacteroides, Proteus, Clostridium	6
Chen et al. [89]	2018	M	1	29	2	11	1	0	Streptococcus Agalactiae, Staphylococcus haemolyticus, Escherichia coli, peptostreptococci, Prevotella corporis	0
Yuan et al. [90]	2018	M	1	62	1	ND	1	ND	Enterococcus avium, Escherichia coli	ND
Katsimantas et al. [91]	2018	M	1	68	2	17	0	0	Enterococcus faecalis, Streptococcus gordonii, Prevotella melaninogenica	0
Althunayyan et al. [92]	2018	F	1	36	2	31	1	0	Escherichia coli, Acinetobacter baumannii	0
Pittaka et al. [93]	2018	F	1	24	> 1	14	ND	ND	ND	0
Taylor et al. [94]	2018	F	1	58	1	ND	1	ND	Bacteroides fragilis, Clostridium ramosum, Gram positive cocci	1
Dos Santos et al. [95]	2018	29 M 11F	40	51.7	1.8	19.6	9	ND	ND	9
Fukui et al. [96]	2018	M	1	85	1	104	1	0	Streptococcus dysgalactiae, Escherichia coli, Staphylococci	0
Kuzaka et al. [97]	2018	13 M	13	59.6	> 1	31.9	0	ND	Enterobacteriaceae, Bacteroides, Parabacteroides, Klebsiella, Staphylococcus, Lactobacillus acidophilus, Escherichia coli	0
Goel et al. [98]	2018	M	1	60	1	14	0	0	ND	0
Ghodoussipour et al. [99]	2018	54 M	54	49.3	3.9	37.5	53	ND	ND	3
Tenório et al. [100]	2018	99 M, 25F	124	50.8	ND	21.7	ND	1	Escherichia coli, Proteus, Klebsiella, Pseudomonas, Staphylococci, Enterococcus, Clostridium	32
Weimer et al. [101]	2017	M	1	55	> 1	90	1	0	Parabacteroides distasonis, Prevotella melaninogenica, Fusobacterium nucleatum, Bacteroides	0
Wähmann et al. [102]	2017	F	1	46	3	ND	1	ND	Streptococci, Enterobacteria, gram +	0
Wang et al. [103]	2017	M	1	61	1	ND	ND	ND	Klebsiella pneumoniae	0
Yücel et al. [104]	2017	11 M, 14F	25	54.3	2.4	21.4	ND	0	ND	1
Üreyen et al. [105]	2017	18 M, 11F	29	51.5	1.8	11.5	17	ND	Escherichia coli, Acinetobacter, Streptococci, Staphylococcus aureus, Pseudomonas, Klebsiella,	6
Dell’Atti et al. [106]	2017	M	1	75	1	28	1	0	ND	0
Yanaral et al. [107]	2017	54 M	54	58.3	1.4	15.3	ND	0	ND	4
Chia et al. [108]	2017	42 M, 17F	59	56	> 1	19	11	ND	Streptococci, Escherichia coli, Prevotella	9
Kordahi et al. [109]	2017	M	1	57	> 1	ND	ND	ND	ND	ND
Hong et al. [110]	2017	18 M, 2F	20	61.8	1.55	36.9	15	0	Escherichia coli, Streptococci, Proteus, Klebsiella pneumoniae, Enterococcus faecium, Pseudomonas aeruginosa, Staphylococcus aureus	5
Sanders et al. [111]	2017	M	1	70	2	ND	1	0	Escherichia coli, P. mirabilis	0
Ferretti et al. [112]	2017	19 M, 1F	20	56	4	31.7	17	4	ND	3
Kumar et al. [113]	2017	M	1	41	2	15	1	0	Streptococcus anginosus, anaerobes, Gram -	0
Ioannidis et al. [9]	2017	20 M, 4F	24	58.9	1	16	18	3	Escherichia coli (11), Klebsiella pneumoniae (3), Pseudomonas aeruginosa (3), Acinetobacter baumannii (2), Proteus mirabilis (2), Providencia stuartii (1)	5
Bocchiotti et al. [114]	2017	M	1	40	3	ND	0	0	Escherichia coli, Streptococcus pyogenes, Prevotella loescheii	0
Choi et al. [115]	2017	F	1	31	1	17	0	0	Streptococcus anginosus, Pseudomonas, Clostridium	0
Sawayama et al. [116]	2017	M	1	66	1	ND	0	0	ND	0
Lauerman et al. [117]	2017	125 M, 43F	168	ND	> 1	ND	92	0	Enterococcus faecalis, Klebsiella pneumoniae, Escherichia coli, Clostridium difficile	6
Smith et al. [118]	2017	M	1	50	> 1	ND	1	0	ND	0
Baek et al. [119]	2017	F	1	57	1	ND	1	ND	ND	0
Huang et al. [120]	2017	M	1	46	1	ND	1	0	ND	0
Morais et al. [121]	2017	12 M, 3F	15	70	ND	32	ND	0	Escherichia coli, Proteus, Staphylococcus aureus, Enterococcus faecalis	4
Okumura et al. [122]	2017	M	1	70	1	39	1	0	Klebsiella pneumoniae, Group G Streptococcus	0
Osbun et al. [123]	2017	ND	165	53.4	1.97	16.6	43	ND	ND	11
Kahn et al. [124]	2017	M	147	52	2.5	19	112	ND	ND	11
Misiakos et al. [125]	2017	47 M, 15F	62	63.7	4.8	19.7	32	0	ND	11
Obi [126]	2017	4 M	4	34.3	1	17.3	0	0	Staphylococcus aureus, Escherichia coli, Pseudomonas aeruginosa, Proteus mirabilis	0
Pernetti et al. [127]	2016	M	1	70	1	21	1	ND	ND	0
Faria et al. [128]	2016	M	1	46	1	4	1	0	ND	0
Ozkan et al. [129]	2016	7 M, 5F	12	62.4	5.7	19.6	ND	0	Polymicrobial flora (6), monomicrobica (6)	0
Yoshino et al. [130]	2016	M	1	64	1	33	1	0	Streptococcus. alpha-emolitico	0
Crowell et al. [131]	2016	M	1	54	3	18	1	0	Rhizopus (zygomycosis)	1
Taken et al. [132]	2016	57 M, 8F	65	52.5	2.5	9.2	13	0	Escherichia coli, Streptococcus, Staphylococcus aureus, Enterobacter, Bacteroides, Pseudomonas aeruginosa, Proteus, Clostridium	6
Wanis et al. [133]	2016	M	1	28	1	14	1	0	ND	0
Sheehy et al. [134]	2016	M	1	48	2	ND	1	0	Polymicrobial flora	0
Sarkut et al. [135]	2016	32 M, 32F	64	57	3	16.6	ND	ND	ND	18
Sinha et al. [136]	2015	F	1	30	1	ND	1	ND	ND	0
Chalya et al. [137]	2015	82 M, 2F	84	34	ND	28	ND	ND	ND	24
Namkoong et al. [138]	2015	M	1	61	1	ND	1	0	ND	0
Mohor et al. [139]	2015	M	1	59	> 1	ND	1	0	ND	0
McCormack et al. [140]	2015	25 M	25	56.6	1.4	ND	3	ND	Polymicrobial flora	5
Tarchouli et al. [141]	2015	64 M, 8F	72	51	3.2	28.7	17	56	Polymicrobial flora (37), Monomicrobial flora (1)	12
Paonam et al. [142]	2015	M	1	65	1	ND	1	0	Escherichia coli, Enterococcus	0
Oguz et al. [143]	2015	34 M, 9F	43	52	> 1	ND	43	0	Polymicrobial flora (Escherichia coli 48%)	6
Asahata et al. [144]	2015	M	1	70	1	ND	0	0	Listeria monocytogenes, Escherichia coli	0
Ye et al. [145]	2015	M	1	47	1	21	0	0	Pseudomonas aeruginosa	0
Danesh et al. [146]	2015	8 M	8	44	> 1	ND	ND	0	Enterococcus, Pseudomonas, Staphylococcus haemolyticus, Proteus, Clostridium	3
Ossibi et al. [147]	2015	M	1	60	1	ND	0	0	ND	0
Grassi et al. [8]	2015	2 M	2	42.5	0.5	ND	2	1	Staphylococcus warneri	1
Sarmah et al. [148]	2015	M	1	68	1	1	1	0	Bacteroides fragilis	1
Papadimitriou et al. [149]	2015	M	1	56	1	90	1	0	Polymicrobial flora	0
Ozsaker et al. [150]	2015	M	1	69	1	ND	0	0	ND	0
Toh et al. [151]	2014	M	1	61	6	ND	1	0	Polymicrobial flora	0
Parry et al. [152]	2014	M	1	48	1	ND	0	0	ND	0
Tena et al. [153]	2014	M	1	73	1	55	1	0	Actinomyces funkei, Clostridium hathewayi, Fusobacterium necrophorum	0
Matilsky et al. [154]	2014	M	1	51	4	30	1	0	Polymicrobial flora	0
Lee et al. [155]	2014	3 M	3	50.7	ND	ND	ND	ND	ND	ND
Di Serafino et al. [156]	2014	M	1	63	1	ND	ND	ND	ND	0
Galukande et al. [157]	2014	2 M	2	35.5	2.5	ND	0	0	ND	0
Tattersall et al. [158]	2014	M	1	61	2	47	1	ND	Escherichia coli	0
Omisanjo et al. [159]	2014	11 M	11	51.9	> 1	22.7	7	0	Klebsiella (10), Escherichia coli, Pseudomonas aeruginosa, no microbes (1)	0
Rubegni et al. [160]	2014	2 M	2	58.5	1	ND	1	0	ND	1
Dinc et al. [161]	2014	M	1	51	> 1	16	0	0	ND	0
Dayan et al. [162]	2014	M	1	27	> 1	ND	0	0	ND	0
Ludolph et al. [163]	2014	3 M	3	48.7	> 1	ND	0	0	ND	0
Ozkan et al. [129]	2014	7 M, 5 F	12	62.4	5.7	19.6	ND	0	Pseudomonas, Acinetobacter, Escherichia coli, Enterococcus, Stafilococcus aureus, Proteus, Corynebacterium, Polymicrobial flora (6)	ND
Shimizu et al. [164]	2014	M	1	74	2	ND	0	0	Proteus vulgaris, Prevotella denticola, Peptostreptococcus species	ND
Ho et al. [165]	2014	F	1	78	1	14	0	0	ND	1
Aslanidis et al. [166]	2014	F	1	23	> 1	ND	1	0	Candida albicans, Staphylococcus epidermidis, Klebsiella pneumoniae	0
D’Arena et al. [167]	2014	M	1	66	1	ND	0	0	ND	0
Perkins et al. [168]	2014	M	1	73	1	ND	0	0	Candida albicans	0
Sliwinski et al. [169]	2014	M	1	24	> 1	ND	1	0	ND	0
Agostini et al. [170]	2014	M	1	64	2	58	1	1	Staphylococcus epidermidis, Proteus mirabilis, Enterococcus faecalis	0
Oymaci et al. [171]	2014	10 M, 6F	16	61.2	4.44	25.5	ND	0	Escherichia coli, Acinetobacter baumannii, Proteus mirabilis, Staphylococcus aureus, Enterococcus	3
Eskitascioglu et al. [172]	2014	76 M, 4F	80	53.5	1.55	34.78	ND	0	Polymicrobial flora (14), Escherichia coli, Staphylococcus aureus, Enterococcus, Acinetobacter baumanii, Staphylococcus epidermidis, Proteus, etc	3
Yilmazlar et al. [173]	2014	81 M, 39F	120	58	3	14.5	48	0	Escherichia coli, Streptococci, Enterococci, Staphylococci, Klebsiella, Pseudomonas, Proteus, fungi	25
Akbulut et al. [174]	2014	M	1	77	1	20	0	0	Escherichia coli	0
Coyne et al. [175]	2014	M	1	48	1	ND	0	0	ND	0
Li et al. [176]	2014	48 M, 3 F	51	49.7	> 1	17	ND	0	Escherichia coli, Streptococcus, Staphylococcus aureus, Pseudomonas, Proteus, Clostridium, Bacteroides	6
Oyaert et al. [177]	2014	M	1	43	1	63	1	0	Atopobium	0
Lee et al. [178]	2013	M	1	47	> 1	ND	0	0	Enterococcus, Enterobacter	0
Abate et al. [179]	2013	M	1	63	1	21	0	0	Enterococcus faecalis, Citrobacter freundii, Pseudomonas aeruginosa, Escherichia coli, Bacteroides fragilis, Bacteroides ovatus	0
Anantha et al. [180]	2013	M	1	59	1	16	1	0	Streptococcus anginosus	0
Benjelloun et al. [181]	2013	44 M, 6F	50	48	2.5	21	11	0	Escherichia coli, Klebsiella	12
Pastore et al. [182]	2013	M	1	60	> 1	34	0	1	Streptococcus A	0
Eray et al. [183]	2013	34 M, 14F	48	53.7	ND	25.3	ND	0	ND	9
Bjurlin et al. [184]	2013	40 M, 1F	41	49	ND	ND	ND	ND	Polymicrobial flora (34), Bacteroides (43.9%), Escherichia coli (36.6%), Prevotella, Streptococci, Staphylococcus aureus	2
Park et al. [185]	2013	M	1	59	> 1	ND	0	0	ND	0
Subramaniam et al. [186]	2013	M	1	80	3	ND	1	0	Escherichia coli, Anaerobes	0
Sabzi Sarvestani et al. [187]	2013	28 M	28	44.6	2.2	17.22	ND	0	Escherichia coli, Bacteroides, Streptococci, Enterococci, Staphylococci, Pseudomonas, Klebsiella, Proteus	10
Katib et al. [188]	2013	20 M	20	55.95	1.7	22.3	1	0	Acinetobacter spp. (most common)	0
Czymek et al. [189]	2013	72 M, 14F	86	57.9	4	52	52	ND	Polymicrobial flora (71), Escherichia coli, Enterococci, Streptococci, Pseudomonas, Staphylococci, etc	14
Akilov et al. [190]	2013	28 M	28	47.1	3.5	24.4	8	0	Monomicrobial flora (18), Staphylococci, Streptococci, Enterobacter, Pseudomonas	0
Bakari et al. [191]	2013	10 M	10	50.5	ND	ND	ND	0	ND	ND
Avakoudjo et al. [192]	2013	ND	72	ND	ND	72	ND	ND	Escherichia coli, Staphylococci, Pseudomonas aeruginosa, Klebsiella	7
Chan et al. [193]	2013	M	1	78	1	ND	1	0	Escherichia coli	0
Chan et al. [194]	2013	M	1	49	15	ND	0	0	Escherichia coli, Streptococci, Arcanobacterium	0
Aliyu et al. [195]	2013	43 M	43	37.82	> 1	28	ND	0	Polymicrobial flora (27)	6
Ozkan et al. [196]	2013	F	1	43	4	ND	1	0	ND	0
Khan et al. [197]	2013	M	1	47	3	ND	1	0	ND	0
Kumar et al. [198]	2013	30 M	30	39.6	2.2	9.7	ND	0	Escherichia coli, anaerobes, Streptococci, Pseudomonas, Staphylococci	6
**Total**		**2463 M** **456 F**	**3423**	**-**	**-**	**-**	**894**	**212**	**-**	**455**

Legend: *M* = male, *F* = female, *h* = hours, *ICU* = intensive care unit, *ND* = not defined.

## Case presentation

**Fig. 1:**
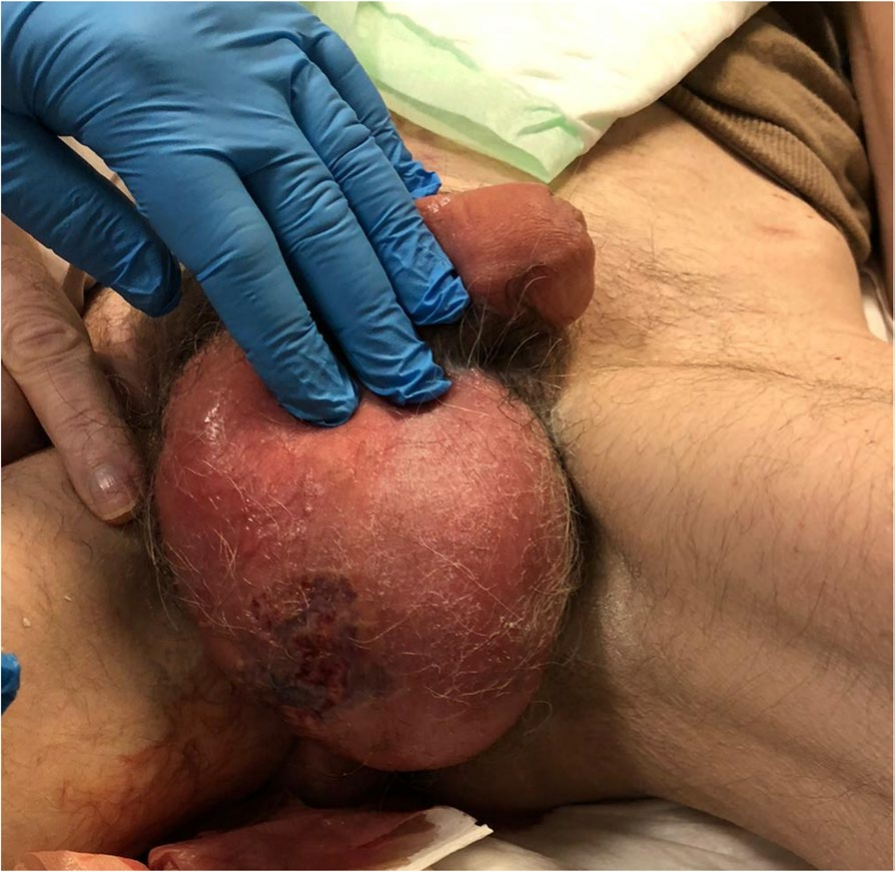
Emergency Department presentation of the case. Clinical presentation with oedema, dyschromia of right hemiscrotum, penis, and perineal region

A 60 years old man affected by diabetes mellitus, Leriche syndrome, with ileostomy after emicolectomy for ulcerative colitis (RCU), was admitted to our Emergency Department with fever, acute pain, oedema, dyschromia of right hemiscrotum, penis, and perineal region (Fig. 1). At the level of the scrotum a visible suppuration was present and vivid pain was evocable.

The blood exams revealed a neutrophilic leukocytosis with 19.1 × 10^9^ white blood cells 83.2% of which neutrophiles, hemoglobin 9.3 g/dl, glucose 314 mg/dl, creatinine 1.2 mg/dl, C-reactive protein 42.7 mg/L, procalcitonin 29.44 ng/ml. The modified Laboratory Risk Indicator for Necrotizing Fasciitis score (LRINEC score) was 7, suspicion for necrotizing fasciitis [61]. The Charlson Comorbidity Index score was of 6, the Fournier’s Gangrene Severity Index was 9 with a risk of death > 75% [199, 200].

The emergency ultrasound exam revealed a marked thickening of the scrotal wall associated with intrafascial anechogen film and multiple hyperechoic spots with posterior echoes as for aerial component.

**Fig. 2:**
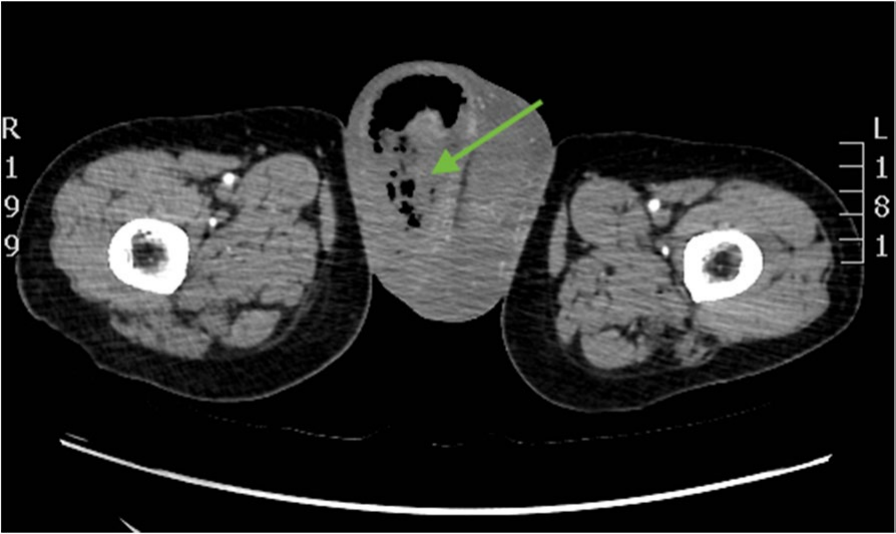
Title. Pre-operative CT-scan. CT-scan revealed air-gas content (green arrow) in the context of the soft and peripheral tissues at the level of the right scrotal lodge. A marked fluid-edematous thickening of the tunics and scrotal walls were present bilaterally but more evident on the right side of the scrotum

Computed Tomography revealed an abundant air-gas content in the context of the soft and peripheral tissues at the level of the right scrotal lodge reached the cutaneous plane at the lower pole and more cranially, further gas was localized at the base of the root of the penis, in the paramedian perineum homolaterally up to floor below the ischium pubic branch (Fig. 2). A marked fluid-edematous thickening of the tunics and scrotal walls were present bilaterally but more evident on the right side of the scrotum.

Intravenous fluid resuscitation and broad-spectrum antibiotics such as Piperacillin/Tazobactam (4.5 gr iv q8h), Imipenem/Cilastatin (500 mg iv q8h) and Daptomycin (700 mg iv q24h) were administered.

**Fig. 3:**
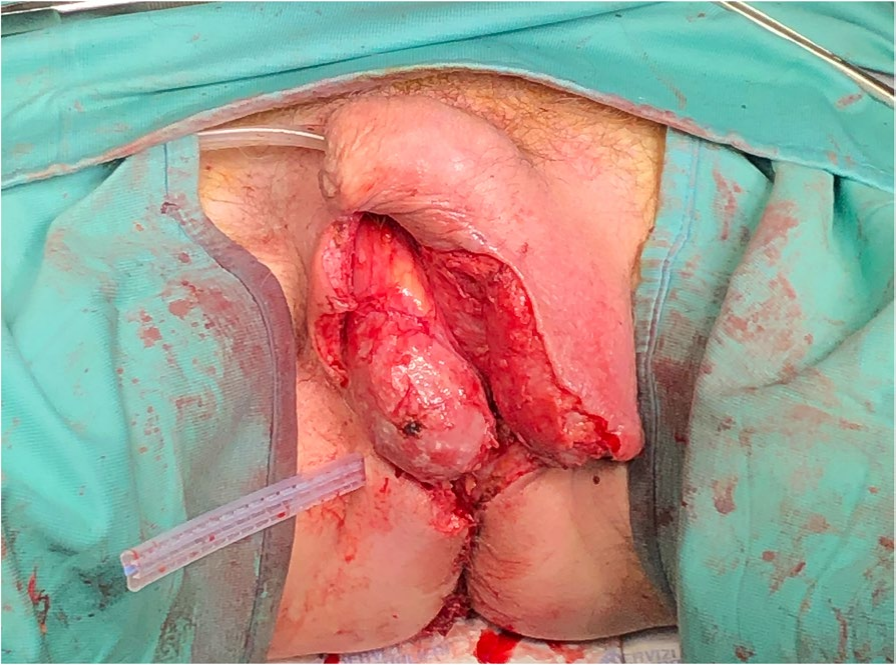
Surgical debridement. Surgical extensive debridement of genitalia and perineal region with exposure of healthy tissue

A prompt surgical debridement of genitalia and perineal region with an accurate necrotic tissue removal up to exposure of healthy tissue was performed (Fig. 3). A Penrose drain was left in place anterior to the rectum where a more destructive debridement was performed. It was removed on the 4^th^ postoperative day after daily withdrawal due to granulated tissue formation. A single blood transfusion was performed for anemia.

Based on intra-operative scrotal ulcer swab, positive for Escherichia coli, Enterococcus faecium, Streptococcus oralis, Candida albicans, Bacteroides fragilis e Staphylococcus lugdunensis, on the 5^th^ postoperative day, the antibiotic therapy was switched to Piperacillin/Tazobactam (4.5 gr iv q8h), Teicoplanin (600 mg iv q24H) and Fluconazole (400 mg iv q24h). Hemocultures and urinocultures were negative.

**Fig. 4:**
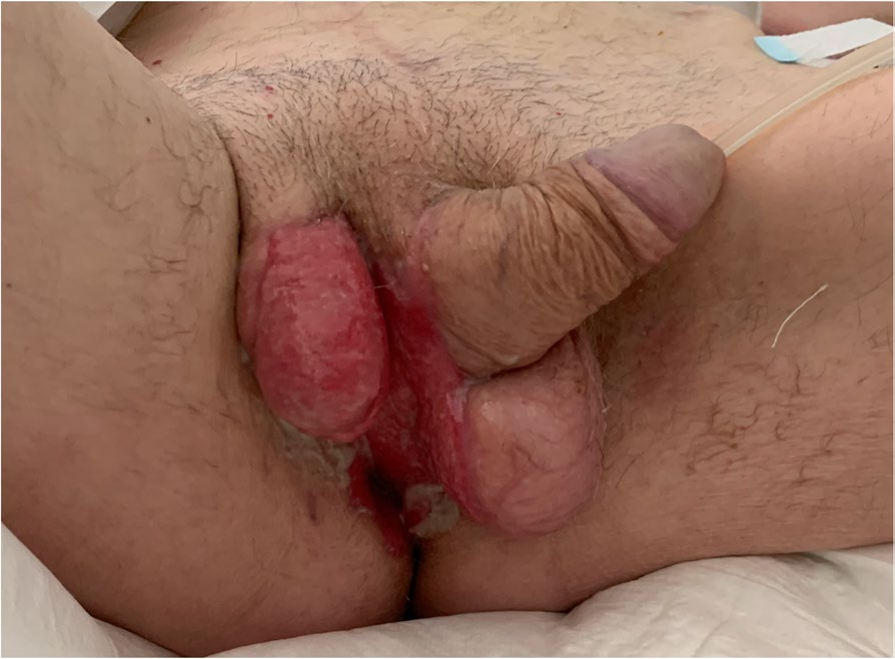
Discharge. Clinical condition at discharge

High-intensity care was carried on in the next days with a bedside daily surgical wound medications with fibrine debridement, normal saline and povidone-iodine solutions irrigation, iodoform and fatty gauze application, until discharge on the 40^th^ postoperative day (Fig. 4).

Plastic surgeons decide to not perform a skin graft due to an excellent wound improvement with local medication. Every 3 days office-based medication with silver dressing, after normal saline and povidone-iodine irrigation and fibrinous tissue debridement, was performed until complete re-epithelialization of the scrotum on the 60^th^ postoperative day.

## Discussion

Predisposing factors to Fournier’s gangrene include all conditions with an impaired micro-circulation and immunosuppression such as diabetes mellitus, obesity, chronic alcoholism, smoking habit, renal and liver failure, malignancies, bowel inflammatory diseases and HIV infection [201–204]. In our case the patient suffered from diabetes, chronic arteriopathy, RCU for which he carried a colostomy following intestinal resection. The presence of a fecal diversion has certainly improved the wound management and therefore promoted its healing, reducing the contamination of the same with fecal material, ensuring a more accurate hygiene of the scrotal and perineal region [183]. The fact that ileostomy was already well established probably allowed to enjoy the benefits described above without exposing the patient to the typical complications of the creation of a neo-stoma, such as parastomal hernia, incisional hernia, colostomy prolapse, necrosis and stenosis which may necessitate additional surgery [183].

Once described as idiopathic, the FG is secondary to aerobic and anaerobic bacterial infection that involves genitalia and perineum and the cause is recognizable in more than 90% of the cases. In most cases the origin site infection is the ano-rectum (30–50%), uro-genitalia (20–40%) and genital surface (20%) [52]. In an immunodeficient host a polymicrobial flora are usually involved with a synergic mechanism of aggressiveness. The latter was present also in our case with several single-management not aggressive pathogens developing a synergism. Polymicrobial infection is reported as cause in 54% of cases [205].

The onset of this necrotizing fasciitis is insidious with up to 40% of cases asymptomatic. When signs and symptoms are the reason of emergency access, they are characterized by genital and perineal regions pain with little to no visible cutaneous damage in the early stage and erythematous and dusky skin, crepitus of subcutaneous tissue, maleodorant and purulent exudates of perineal and genital regions [206].

A successful management of the Fournier’s gangrene is challenging. The risk of death in about 20% of patients makes FG an emergency health condition [68, 99]. Fluid resuscitation for adequate systemic perfusion, empiric intravenous broad-spectrum antibiotic therapy to reduce the risk of septic shock and a prompt extensive surgical debridement ensured an improvement in prognosis in accordance with current guidelines [207]. The surgery plays a cardinal role because a delay in surgical debridement is associated with a significant increase in mortality [208]. From the review of the literature, a risk of death up-to-date is of 14.3% (Table).

In addition, the necrotizing fasciitis could benefit from hyperbaric oxygen therapy (HBOT) to reduce the spread of anaerobic germs, from the vacuum-assisted closure (VAC) that can be used to promote wound healing physiologically reducing the need for reconstructive surgery with skin graft in the setting of a personalized medicine [206, 209–211]. HBOT has been related to a better wound control as an adjuvant treatment by promoting wound healing. It acts as bactericide and bacteriostatic especially over anaerobic bacteria, almost always involved in this necrotizing fasciitis. HBOT increases local circulation and tissue oxygenation which prevents the progression of necrosis; furthermore, HBOT seems have synergism with certain antibiotics [18, 45, 209]. In our case the patient hospitalization was long due to the difficulties related to the COVID pandemic era, the choice to not perform a skin graft and the need for daily medications in order to obtain a natural restitutio of the lesion as possible. This type of management made it possible to avoid the use of common tools for resolving Fournier's gangrene such as HBOT, VAC and surgical graft. In our hospital there is not the HBOT so it would have been necessary to transfer the patient to another hospital and one of the COVID-19 pandemic period problem was the patient’s displacement and outpatient hospital management. For all these reasons we decided for a conservative inpatient management.

## Conclusions

FG is burdened of high risk of death and a prompt multimodal approach is mandatory. This necrotizing fasciitis also needs a post-operative rigid management to reduce a risk of relapse and allow a complete restoration. In our case, for reason of necessity, an immediate multimodal approach and a daily cleaning of the surgical wound allowed to obtain its complete restoration avoiding HBOT, VAC or surgical graft without foregoing optimal outcomes.

## Data Availability

All data generated or analysed during this study are included in this published article.

## Abbreviations

FG: Fournier’s gangrene
HBOT: Hyperbaric oxygen therapy
RCU: Ulcerative colitis
VAC: Vacuum-assisted closure
iv q8h: Intravenously every 8 h
